# A coarse-grained biophysical model of sequence evolution and the population size dependence of the speciation rate

**DOI:** 10.1016/j.jtbi.2015.04.027

**Published:** 2015-08-07

**Authors:** Bhavin S. Khatri, Richard A. Goldstein

**Affiliations:** a The Francis Crick Institute, Mill Hill Laboratory, The Ridgeway, London NW7 1AA, UK; b Division of Infection & Immunity, University College London, London WC1E 6BT, UK

**Keywords:** Dobzhansky Muller incompatibilities, Evolution, Genotype phenotype map, Sequence entropy, Free fitness

## Abstract

Speciation is fundamental to understanding the huge diversity of life on Earth. Although still controversial, empirical evidence suggests that the rate of speciation is larger for smaller populations. Here, we explore a biophysical model of speciation by developing a simple coarse-grained theory of transcription factor-DNA binding and how their co-evolution in two geographically isolated lineages leads to incompatibilities. To develop a tractable analytical theory, we derive a Smoluchowski equation for the dynamics of binding energy evolution that accounts for the fact that natural selection acts on phenotypes, but variation arises from mutations in sequences; the Smoluchowski equation includes selection due to both gradients in fitness and gradients in sequence entropy, which is the logarithm of the number of sequences that correspond to a particular binding energy. This simple consideration predicts that smaller populations develop incompatibilities more quickly in the weak mutation regime; this trend arises as sequence entropy poises smaller populations closer to incompatible regions of phenotype space. These results suggest a generic coarse-grained approach to evolutionary stochastic dynamics, allowing realistic modelling at the phenotypic level.

## Highlights

• We explore a biophysical mechanism of speciation.
• We develop a novel coarse-grained stochastic dynamics of sequence evolution.
• Analytical results show that hybrid binding energies diffuse neutrally.
• Sequence entropy and drift poise common ancestors closer to incompatible regions.
• So as population size decreases hybrid incompatibilities arise more quickly.

## 1 Introduction

Speciation underlies the diversity of life on Earth today. Yet the detailed genetic mechanisms by which distinct species arise are still largely not understood. Darwin (1859), despite the title of his magnus opus, struggled to understand how natural selection could give rise to hybrid inviability or infertility. If the hybrid inviability were due to a single locus, how could two species evolve from a common ancestor, as one of these species would have to evolve past an inviable heterozygotic state. A resolution came with the understanding that epistatic (non-linear) interactions between different loci can give rise to the so-called Dobzhansky–Muller incompatibilities (DMI) between independently evolving lineages (Dobzhansky, 1936; Muller, 1942; Bateson, 1909; Gavrilets, 2004). For example, two lineages evolving independently through geographic isolation (allopatric evolution) from a common ancestor *ab* can fix the genotypes *aB* and *Ab*, yet the hybrid genotype *AB* may be inviable. Through a similar mechanism incompatibilities can arise in polygenic systems, where the effective contribution to fitness of the many loci coding a quantitative trait fitness is epistatic. Even if the loci contribute additively to the trait, stabilising selection (usually modelled as quadratic) on a trait value induces epistasis. Populations diverge, under the action of drift, by shifting between different stable equilibria that encode the same optimal trait value, but with different allelic combinations (Wright, 1935a,b); when combined in hybrids this can lead to hybrid incompatibilities (Barton, 1989). Field data (Coyne and Orr, 2004; Mayr, 1963) and specific introgression studies (Wu and Beckenbach, 1983; Vigneault and Zouros, 1986) suggest that the most dominant form of speciation involves the generation of hybrid incompatibilities in geographically isolated populations with no or very little gene flow.

The development of quantitative models that can predict speciation rates will allow better understanding of the different factors that maintain bio-diversity along with the processes of extinction and environmental change (Coyne and Orr, 2004; Rosenzweig, 2001). An important aspect of such models is the dependence of speciation rate on population size. Although, the question of a population size dependence of the rate of speciation has received little empirical attention and there have yet to be any definitive studies, there is indirect evidence that the rate of speciation is higher in smaller populations (Santos and Salzburger, 2012; Mayr, 1970; Glor et al., 2004), including the large species diversity of fish in the East African Great Lakes (Owen et al., 1990) compared to marine animals (Mayr, 1970, 1954; Rubinoff and Rubinoff, 1971) and birds (Fitzpatrick, 2004) which have large ranges and populations sizes, and the population size dependence observed in net diversification rates inferred from phylogenetic trees (Coyne and Orr, 2004; Nee, 2001; Barraclough and Nee, 2001). Strikingly, although cichlid fishes in Lake Malawi, whose effective population sizes are of order 100–10 000 (Oppen et al., 1997; Fiumera et al., 2000), develop reproductive isolation within 1–10 Myr after divergence (Stelkens et al., 2010), domestic chickens (*Gallus gallus*) can still hybridise with helmeted guineafowl (*Numida meleagris*) after roughly 55 Myr divergence (Cooper and Penny, 1997), potentially reflecting the large effective population size of domestic chickens estimated to range between 10^5^ and 10^6^ (Sawai et al., 2010).

Models of speciation that require positive selection to drive divergence are unlikely to be able to explain these trends as larger populations take less time to fix beneficial mutants and so evolve more quickly (Gavrilets, 2003). Founder event or peak shift models where reproductive isolation arises when a small population passes through a fitness valley could explain this trend, as the rate of valley crossing increases at small population sizes (Lande, 1979, 1985; Barton and Charlesworth, 1984; Barton and Rouhani, 1987). However, these models require a small fitness valley to give speciation on realistic timescales, meaning that the reproductive isolation this model seeks to explain is generally destroyed. In the strong mutation regime (mutation rate large relative to the inverse population size), polymorphisms will be common, and the larger variation found in larger populations is predicted to result in a slower average substitution rate, reducing the rate of speciation (Gavrilets, 1999; Nei et al., 1983). Polygenic models of divergence of additive traits under stabilising selection, also in the strong mutation regime, predict that smaller populations can shift between stable equilibria more quickly, leading to more rapid isolation (Barton, 1989). More recently, sequence-level simulations of protein–DNA binding similar to the model we examine here, showed in the intermediate to strong mutation regime, that hybrid fitness decayed more rapidly for smaller populations (Tulchinsky et al., 2014); however, the underlying mechanism or growth of DMIs was not explored. Despite these results in the strong mutation regime, many traits involved in speciation are found to be monogenic or oligogenic (involving only one or a few loci) (Orr, 2001) and so are expected to arise in monomorphic populations in the weak mutation regime. In this respect, Orr constructed a model that considers the combinatorics of how potential incompatibilities grow between two independent lineages in the weak mutation regime. For pair-wise interactions between loci this growth is quadratic in the number of substitutions by which they are separated (Orr, 1995; Orr and Turelli, 2001); however, the model assumes that populations diverge neutrally and so predicts no population size dependence. To summarise, although theory predicts that in the strong mutation regime we would expect a slower rate of accumulation of DMIs for larger populations, there are no theories of speciation that predict this population size effect in the very relevant weak mutation regime.

In this paper, we examine the process of how incompatibilities arise in allopatry for a biophysical model of a transcription factor binding to DNA by developing a coarse-grained model of how the transcription factor protein and DNA sequences co-evolve within a stochastic dynamics framework. Our key innovation is to develop a general equation of phenotypic evolution in the weak mutation regime, which accounts for the fact that selection acts on phenotypes, but variation in phenotype arises from mutations in sequence through the mapping of genotype to phenotype. In particular, we need to include the number of sequences corresponding to a particular phenotype, the log of which we call the “sequence entropy” in analogy to statistical mechanics entropy. This approach normally gives rise to an often intractable master equation. By considering the continuous limit, however, we can convert the master equation into a diffusion equation called the Smoluchowski equation, which includes selection, sequence entropy, and random drift. By including the effects of sequence entropy, the stochastic dynamics framework we present allows investigation of the effect of population size on evolution in the weak mutation regime, including its role in speciation dynamics. Our work differs from previous diffusion-based models of phenotypic evolution, such as Lande (1976), by considering a generic genotype–phenotype map and also in focusing on the weak mutation regime where we can ignore polymorphisms and restrict our attention to movement between monomorphic genotypes. What we find is a picture of speciation different from that of the Orr model, in that it features a latency in the development of DMIs as hybrid populations need a finite time to reach incompatible regions of phenotype space. Importantly, the model predicts a higher rate of speciation in smaller populations in the weak mutation regime, providing an explanation for the trend seen in the observations described above.

Gene expression divergence has been shown to be a major factor in driving differences between species (King and Wilson, 1975; Wolf et al., 2010; Wray, 2007; Wittkopp et al., 2008), and there is direct evidence of speciation driven by the evolution of genes related to transcription factors in *Drosophila* (Ting et al., 1998; Brideau et al., 2006). Thus the binding of transcription factors to DNA to control gene expression is arguably one of the most important co-evolving systems for organisms and crucial for their correct development, making them an ideal case study for a biophysical model of speciation. However, despite our focus on transcription factor binding, the model is in fact very generic and could form the basis for the co-evolution of a number of interacting macromolecules including protein–protein interactions, antibody–antigen interactions, or the interaction of genes expressed by nucleus and mitochondria.

We first derive a diffusion equation (Smoluchowski equation) for studying the coarse-grained stochastic evolutionary dynamics of co-evolving sequences, and then adapt this model to the case of two interacting genes represented by the binding of a transcription factor to a region of DNA. We then consider two populations evolving independently from a common ancestor, and consider the viability of reproductive crosses between these populations.

## 2 A Smoluchowski equation for evolutionary stochastic dynamics

Natural selection acts on phenotypes. In general, however, many genotypes code for the same phenotype (Fontana, 2002; Force et al., 1999; Gerland and Hwa, 2002; Berg et al., 2004; Khatri et al., 2009; Mustonen and Lassig, 2005) exemplified by proteins with near identical structures but divergent sequences or different RNA sequences that give rise to the same RNA secondary structure. This can give rise to a bias in evolution towards phenotypes corresponding to a larger number of sequences. A powerful approach to dealing with this degeneracy is through the concept of sequence entropy, representing the (log) number of sequences encoding a given phenotypic state, in analogy to the concept of entropy in statistical mechanics. By including sequence entropy explicitly in the formulation, we can develop a novel coarse-grained approach to evolutionary dynamics that allows study of changes that occur at the phenotypic level where selection acts, while still accounting for the fact that variation or mutations arise at the sequence level.

We consider the weak mutation regime where $n\mu_{0}N_{e}\ll 1$, where *μ*_0_ is the effective base pair mutation rate, *n* is the number of all contributing sites or base pairs, and *N*_*e*_ is the effective population size. In this regime, rare mutations are sequentially fixed or eliminated in an otherwise monomorphic population (Wright, 1931). Under this condition, Iwasa (1988) and Sella and Hirsh (2005) showed that the probability of observing genotype g is given by a Boltzmann-like distribution $p(g)=(1/Z)e^{\nu F(g)}$, where F(g)=lnW(g) is the log fitness (or equivalently the additive Malthusian fitness) of genotype g,ν is proportional to the effective population size (analogous to the inverse temperature of the canonical ensemble from statistical mechanics), and *Z* is the normalisation factor (or partition function) that makes sure the probabilities sum to one. Here we assume that either the environment is fixed or that the fitness is an average over the variation in phenotypes produced by environmental variation or stochasticity. For the rest of the paper we will assume a diploid Wright–Fisher process, where $\nu =2(2N_{e}-1)\approx 4N_{e}$.

We wish to consider distributions of phenotypes rather than genotypes. Assuming a genotype to phenotype map ξ=Ξ(g) that maps each genotype g to corresponding phenotype ξ, we can consider the number (or degeneracy) of genotypes Ω(ξ) that map to any specific phenotype ξ. We can then sum over genotypes to give the probability of observing a specific phenotype (Barton and Coe, 2009):

(1) $$p(\xi )=\frac{1}{Z}\Omega (\xi )e^{4N_{e}F(\xi )}=\frac{1}{Z}e^{4N_{e}\Phi (\xi )}.$$

where the effective potential function of the evolutionary dynamics is given by the “free fitness” (in analogy to the free energy in statistical mechanics),

(2) $$\Phi (\xi )=F(\xi )+\frac{1}{4N_{e}}S(\xi ),$$

where the sequence entropy *S* is given by

(3) S(ξ)=ln(Ω(ξ))

A similar entropy was used to understand the equilibrium solutions of polygenic traits under the balance of stabilising selection and mutation (Barton, 1989). Eq. (2) shows that the equilibrium distribution of phenotypes is in general described by a balance between increasing fitness and increasing sequence entropy (Iwasa, 1988; Sella and Hirsh, 2005).

We expect that in the monomorphic weak mutation regime, stochastic evolutionary dynamics will give rise to diffusion in phenotype space with diffusion constant $\mu =n\mu_{0}$, combined with directed motion driven by gradients in the free fitness function with respect to changes in phenotype ξ.^1^ Note that this gradient includes derivatives of fitness as well as sequence entropy. As we are dealing with a stochastic system, we will describe the time evolution of the probability distribution of phenotypic states p(ξ). The flux J of probability in phenotype space can be written as (Gardiner, 2009):

(4) $$J=-\frac{1}{2}\mu \nabla p(\xi )+\frac{1}{\zeta }p(\xi )\nabla \Phi (\xi )$$

where *ζ* is a coefficient representing the strength of evolutionary change in response to the gradient in free fitness, and the factor of a $\frac{1}{2}$ for the mutation rate comes from converting from a discrete random walk to a continuous one. We can determine the value of *ζ* by considering the equilibrium state; strictly in the monomorphic regime ($n\mu_{0}N_{e}\ll 1$), when the distribution of phenotypes is in equilibrium, as represented by Eq. (1), we would expect the flux to be zero. This condition is satisfied as long as

(5) $$\zeta =\frac{1}{2N_{e}\mu },$$

which is the evolutionary equivalent of the Einstein relation that relates the friction constant to the diffusion constant of a Brownian particle (Einstein, 1905). Using the continuity equation, which guarantees that probability is locally conserved as it flows from point to point, $\partial_{t}p(\xi )=-\nabla \cdot J(\xi )$, we generate the Smoluchowski equation in its final form:

(6) $$\frac{\partial p}{\partial t}=\frac{1}{2}\mu \nabla \cdot (\nabla p(\xi )-4N_{e}p(\xi )\nabla \Phi (\xi )).$$

In Appendix A, we derive the same diffusion equation more rigorously in one-dimension using the Kramers–Moyal expansion of a generalised master equation.

In physics, a diffusion equation with fluxes in probability (Eq. (4)) which are proportional to the gradient of a potential function, and whose strength is related to the strength of stochastic interactions is the Smoluchowski equation. Here, the stochastic interactions correspond to genetic drift, whose relative strength diminishes with increasing population size.

The Smoluchowski equation is equivalent to the set of stochastic differential equations representing the time dependence of the values of individual traits (Gardiner, 2009; van Kampen, 1981)

(7) $$\frac{d\xi_{i}}{dt}=2N_{e}\mu \frac{\partial \Phi (\xi )}{\partial \xi_{i}}+\eta_{i}(t),$$

where *i* corresponds to the ith trait of ξ and where *η*_*i*_ is a white noise Gaussian process with moments $\langle \eta_{i}(t)\rangle =0$ and $\langle \eta_{i}(t)\eta_{j}(t^{\prime })\rangle =\mu \delta_{\mathrm{ij}}\delta (t-t^{\prime })$. Eq. (7) is a generalisation of the Ornstein–Uhlenbeck process for phenotypic evolution described by Bedford and Hartl (2009), but for an arbitrary free fitness landscape and including the correct population size dependence of the strength of the drift term via the Einstein relation Eq. (5). This set of stochastic differential equations is similar to those used by Nei et al. (1983), but includes the effects of sequence entropy. Eq. (6) is also similar to the phenotypic diffusion approximation developed by Lande (1976), but here represents the longer timescale stochastic exploration of phenotype space due to the sequential fixation of mutations, rather than the shorter scale dynamics of co-existing and competing alleles.

## 3 Coarse grained biophysical model of protein–DNA binding

We aim to use this stochastic dynamics to study speciation for a co-evolving pair of loci representing the binding of a transcription factor (TF) to a region of DNA corresponding to the TF binding site (TFBS). The key to this approach is the calculation or specification of a sequence entropy function that represents the mapping between genotype and phenotype. The two-state approximation (von Hippel and Berg, 1986; Gerland et al., 2002) for transcription factor binding assumes that amino acid base pair hydrogen-bonding binding energies are approximately additive and that each non-optimal interaction increases the energy of binding by approximately the same amount. The rationale for this model is the observation that there tend to be preferred nucleotides for each amino acid which hydrogen bond well, while non-preferred nucleotides cause a large destabilisation by blocking the formation of water–DNA hydrogen bonds. Rather than considering a more realistic quaternary alphabet, for simplicity we only consider whether the amino-acid DNA nucleotide pair is favourable or not by replacing DNA and amino acid sequences by binary strings $g_{1}$ and $g_{2}$ of length ℓ and letting the binding energy be proportional to the number of mismatches (Hamming distance) *r* between them, so ΔG=εr where

(8) $$r=(g_{1}-g_{2})\cdot (g_{1}-g_{2}),$$

The binding energy phenotype is additive in each amino-acid nucleotide position, analogous to polygenic models of quantitative traits where trait values are assumed additive with respect to each contributing loci (Wright, 1935a,b; Barton, 1989). In the model described here, however, the binding energy is a non-linear function of the sequences at each loci, as is clear from Eq. (8). Epistasis arises directly from the molecular interactions, as whether or not a position at the binding interface is mismatched depends on a comparison between the amino acid and nucleotide in opposing positions, which each come from different loci.

Relating the fitness of an organism to the binding energy of a TF to its binding site is in principle very complicated and is controlled by a number of factors dependent on the particular context of the gene being regulated. Genomewide studies of the distribution of binding energies for given TFs in *E. coli* (Mustonen and Lassig, 2005) and yeast (Mustonen et al., 2008; Haldane et al., 2014) suggest that maximum fitness arises for strongest binders when the number of mismatches between TF and TFBS is minimised, with a non-linear dependence for increasing binding energy with negative curvature; although such studies relate to an average over different fitness contexts for each binding site, they are indicative of a fitness landscape that favours the fewest mismatches. Binding to the TFBS must compete with non-specific binding to other sites in the genome suggesting that fitness would be a sigmoidal function of the binding energy (Gerland et al., 2002). For simplicity, we represent this as a quadratic log fitness function (corresponding to a Gaussian fitness landscape) up to a specific Hamming distance *r*^⁎^. $r>r^{⁎}$, corresponding to $\Delta G>\Delta G^{⁎}=\varepsilon r^{⁎}$, represents an inviability boundary for binding energies, when non-specific binding to all other sites becomes more thermodynamically favourable than binding to the TFBS. The hard limit at *r*^⁎^ also allows us to calculate how the probability of an incompatibility increases with divergence time and make comparison to the work of Orr (Orr and Turelli, 2001); we would not expect any scaling of incompatibilities with divergence time to be too sensitive to the exact choice of threshold. The fitness is then

(9) $$F\left(r\right)=\left\{\begin{array}{l} -\frac{1}{2}\kappa_{F}r^{2}\;\mathrm{for}\;r\leq r^{⁎}\\ -\infty \;\mathrm{for}\;r>r^{⁎} \end{array}\right)$$

where *κ*_*F*_ is the curvature of the fitness landscape and biologically, roughly corresponds to the strength of selection of this trait; as *κ*_*F*_ decreases the fitness landscape becomes more shallow, and so for a fixed effective population size the landscape becomes more neutral.

The number of sequences corresponding to a given Hamming distance *r* is simply proportional to the number of ways of placing a mismatch amongst a pair of sequences, Ωr=(ℓr)≈2ℓ2/πℓexp(−(2/ℓ)(r−ℓ/2)2) when ℓ is large. So according to Eq. (3), to a good approximation the sequence entropy is quadratic in Hamming distance *r*:

(10) $$S(r)=-\frac{2}{\ell }{\left(r-\ell /2\right)}^{2}+\mathrm{const}.$$

We see that entropy is maximised for r≈ℓ/2, reflecting the fact that matches and mismatches are equally likely.

The master equation governing the dynamics with entropy *S*(*r*) is in general difficult to handle analytically. Instead, we take advantage of the observation that at sufficiently small population sizes the population size is larger than the typical change in fitness between discrete states so that many mutations are nearly neutral (Sawyer and Hartl, 1992; Bustamante et al., 2002; Eyre-Walker et al., 2006; Piganeau and Eyre-Walker, 2003; Yampolsky et al., 2005). As shown below, in this limit the discrete dynamics can be accurately approximated by the effective stochastic dynamics described by Eq. (6) or Eq. (7) by replacing each sequence $g_{i}$ with a continuous variable *x*_*i*_ and equating the Hamming distance *r* with a distance-like variable $\xi =|x_{1}-x_{2}|$, which is proportional to the binding energy with scaling constant *ε*; as the value of *ε* will not affect the qualitative behaviour, we set ε=1 so that *ξ* represents the binding energy. This is the key novelty of our approach that allows us to model coarse-grained evolutionary dynamics at a phenotypic level, at which natural selection acts, while accounting for the fact that variation arises at the sequence level. This approximation involves replacing a high-dimensional space of possible binary sequences with a one-dimensional continuous space, where the effect of high-dimensionality is included through the explicit consideration of sequence entropy through the entropy function in Eq. (10); as selection only acts on phenotypes, our approach ensures that we produce a continuous distribution of phenotypes from our continuous variables *x*_*i*_ that closely approximates the discrete distribution from real sequences $g_{i}$.

**Fig. 1:**
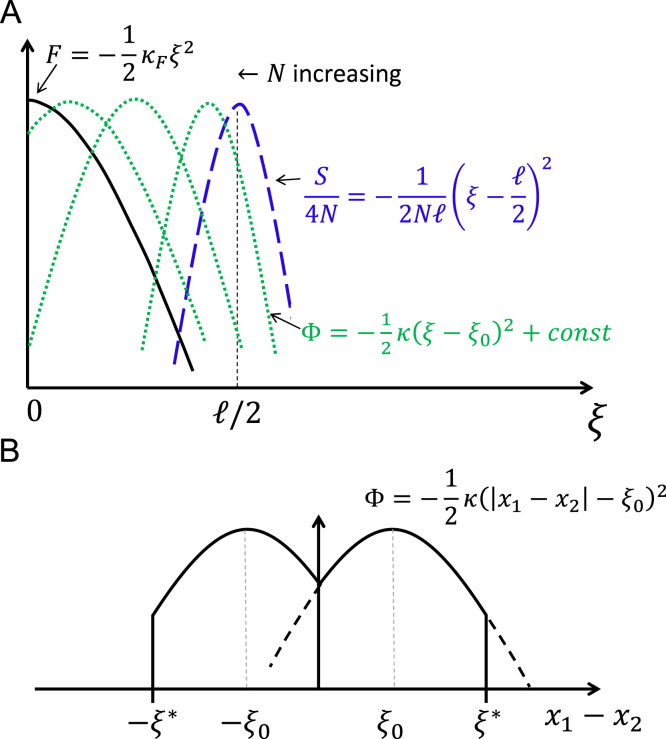
(A) Schematic plot of free fitness landscape Φ(ξ) as a function of population size (with $\xi^{⁎}=\infty$). Free fitness is given by $\Phi (\xi )=F(\xi )+(1/4N_{e})S(\xi )$, so when both fitness and sequence entropy are quadratic, it is also quadratic with maximum given by $\xi_{0}=1/2\kappa N_{e}$, where $\kappa =\kappa_{F}+1/\ell N_{e}$ is the sum of curvatures due to fitness and entropy. For large population sizes ($2\kappa_{F}N_{e}\gg 1/\ell$), fitness dominates, so that Φ(ξ)→F(ξ) and the most probable phenotype $\xi_{0}\to 0$ (black). For small population sizes sequence entropy dominates, so that $\Phi (\xi )\to (1/4N_{e}S)(\xi )$ and the most probable phenotype $\xi_{0}\to \ell /2$ (blue). For intermediate population sizes, there is balance between fitness and sequence entropy, shown by quadratic curves with maxima $0<\xi_{0}<\ell /2$ (green) that shift to the left for increasing population size. It is this mechanism that shifts common ancestors closer to the inviability boundary that is responsible for the faster growth of DMIs at small population sizes. (B) Free fitness landscape as a function of $x_{1}-x_{2}$ is doubled welled with a cusp barrier at $x_{1}=x_{2}$. The approximation that leads to Eq. (16) amounts to assuming the landscape is single peaked with maximum at *ξ*_0_, as shown by the dotted line. (For interpretation of the references to colour in this figure caption, the reader is referred to the web version of this paper.)

Making the substitutions $g_{i}\to x_{i}$ and r→ξ and the sequence entropy given by Eq. (10) the free fitness is given by

(11) $$\Phi \left(\xi \right)=\left\{\begin{array}{l} -\frac{1}{2}\kappa {\left(\xi -\xi_{0}\right)}^{2}\;\mathrm{for}\;\xi \leq \xi^{⁎}\\ -\infty \;\mathrm{for}\;\xi >\xi^{⁎} \end{array}\right)$$

to within an irrelevant constant, where the curvature in the free fitness landscape

(12) $$\kappa =\kappa_{F}+\frac{1}{\ell N_{e}},$$

is the sum of the curvatures due to fitness and sequence entropic potentials, and

(13) $$\xi_{0}=\frac{1}{2\kappa N_{e}}=\frac{1/2}{\kappa_{F}N_{e}+1/\ell }$$

is the phenotype with maximum free fitness (see Fig. 1A) corresponding to the most probable phenotype. Note that as the equilibrium probability density $p(\xi )=(1/Z)e^{4N_{e}\Phi (\xi )}=(1/Z)e^{-2N_{e}\kappa {\left(\xi -\xi_{0}\right)}^{2}}$ is Gaussian, the standard deviation or width of populations on the free fitness landscape is approximately

(14) $$\Delta \xi \sim \frac{1}{\sqrt{4\kappa N_{e}}}.$$

Although the fitness landscape is independent of population size, the free fitness landscape has an explicit dependence on population size due to the contribution from sequence entropy. In particular, Eqs. (12) and (13) show that in the limit of large population sizes, where $\kappa_{F}\gg 1/N_{e}\ell$, we have a simple stabilising fitness landscape with a population scaled strength of selection $4N_{e}\kappa =4N_{e}\kappa_{F}$, which increases with increasing effective population size, as we would expect from standard theory when selection dominates. Conversely, for small population sizes ($\kappa_{F}\ll 1/N_{e}\ell$), the population scaled curvature in free fitness becomes $4N_{e}\kappa =4/\ell$ which is independent of population size, again as we would expect from standard neutral theory. The free fitness in this quadratic form thus relates to previous studies of quadratic fitness with an optimum trait value (Kimura, 1965; Lande, 1975; Turelli, 1984; Barton and Turelli, 1987), although these studies mainly focussed on the maintenance of variation under stabilising selection in the strong mutation regime.

From Eq. (7), we can write down a pair of stochastic differential equations describing the dynamics of these sequence-like variables in a quadratic truncated landscape:

(15) $$\frac{dx_{1}}{dt}=2N_{e}\mu \frac{\partial \Phi (x_{1},x_{2})}{\partial x_{1}}+\eta_{1}(t),\frac{dx_{2}}{dt}=2N_{e}\mu \frac{\partial \Phi (x_{1},x_{2})}{\partial x_{2}}+\eta_{2}(t),$$

where the free fitness is as given by Eq. (11) and shown schematically by the solid black line in Fig. 1B. In this form the equations are not easily solved analytically and so we next make some approximations to make them tractable; in the results section we compare the numerical integration of the above equations with the approximate analytical theory.

Although the free fitness landscape has a single peak with respect to $\xi =|x_{1}-x_{2}|$ (Fig. 1A), the free fitness as a function of $x_{1}-x_{2}$ has two maxima corresponding to $x_{1}-x_{2}=\pm \xi_{0}=\pm 1/2\kappa N_{e}$ with a cusp valley at $x_{1}-x_{2}=0$ as shown in Fig. 1B. We are mostly interested in the short time evolutionary behaviour; as demonstrated below, it is unlikely that the population will sample either the inviability threshold $x_{1}-x_{2}=\xi^{⁎}$ or the cusp at $x_{1}-x_{2}=0$. This allows us to assume that each lineage evolves in a single peak quadratic free fitness landscape shown by the dotted lines in Fig. 1B. We can then set $\xi =x_{1}-x_{2}$, from which it follows:

(16) $$\frac{dx_{1}}{dt}=-2N_{e}\kappa \mu (x_{1}-x_{2})+\mu +\eta_{1}(t),\frac{dx_{2}}{dt}=-2N_{e}\kappa \mu (x_{2}-x_{1})-\mu +\eta_{2}(t),$$

where the characteristic relaxation rate of the system is given by $2N_{e}\kappa \mu$. With this approximation $\langle \xi \rangle =\xi_{0}$; in reality the free fitness landscape, as shown in Fig. 1A, is effectively truncated for ξ<0 and $\xi >\xi^{⁎}$, so strictly $\langle \xi \rangle \neq \xi_{0}$; however, as we show below this small error has little effect on the dynamics, particularly for short times (μt≪1). It is straightforward to take the Laplace transform of these equations, solve the resulting matrix equation to give solutions in Laplace space and find the inverse Laplace transform to give

(17) x(t)=Jx(0)+14Neκ(1−e−4Neκμt)(1−1)+∫0tJ(t−t′)η(t′)dt′

where $x={\left(x_{1},x_{2}\right)}^{T}$, $\eta ={\left(\eta_{1},\eta_{2}\right)}^{T}$, the matrix J is given by

(18) $$J=\frac{1}{2}(\begin{array}{c} 1+e^{-4N_{e}\kappa \mu t}\;1-e^{-4N_{e}\kappa \mu t}\\ 1-e^{-4N_{e}\kappa \mu t}\;1+e^{-4N_{e}\kappa \mu t} \end{array}),$$

and the integral of the vector above is an element by element operation.

## 4 Independently evolving populations and the probability of Dobzhansky–Muller incompatibilities

Let us imagine two separate allopatric populations, each containing the two interacting genes. The two lineages are characterised by $x=\{x_{1},x_{2}\}$ and $x^{\prime }=\{x_{1}^{\prime },x_{2}^{\prime }\}$; at the point of allopatriation $x^{\prime }(t=0)=x(t=0)$. For simplicity, we assume that the binding energy of the common ancestor is $\xi (0)=\xi_{0}$ the most probable value of the binding energy Eq. (13). (In reality it would be described by a distribution $p(\xi )=e^{4N_{e}\Phi (\xi )}/Z$ of initial conditions, which is centred around *ξ*_0_.) The two populations then evolve independently as described by Eq. (16) with solutions x(t) and $x^{\prime }(t)$ given by Eq. (17).

We assume no linkage between loci, so that there is free recombination between loci in hybrids; note, however, that as we are in the weak mutation limit, recombination is irrelevant for evolution within each lineage. Speciation occurs when cross-mating between the two diverged lineages result in incompatible sets of interacting alleles. In the current model, these consist of combining *x*_1_ with $x_{2}^{\prime }$ or $x_{1}^{\prime }$ with *x*_2_. If we let $w=x_{1}-x_{2}^{\prime }$ and $w^{\prime }=x_{1}^{\prime }-x_{2}$, where |w| and $\left|w^{\prime }\right|$ are the binding energies of the hybrids, it is straightforward to show using Eqs. (17), (18) and $\xi (0)=\xi_{0}$ that

(19) $$\langle w\left(t\right)\rangle =\langle w^{\prime }\left(t\right)\rangle =\langle \xi \left(t\right)\rangle =\langle \xi^{\prime }\left(t\right)\rangle =\frac{1}{2\kappa N_{e}}$$

which is the most probable value of binding energy Eq. (13).^2^

If we define the vector $w={\left(w,w^{\prime }\right)}^{T}$, we find that the covariance matrix $\Sigma ={\left(w-\langle w\rangle \right)}^{T}(w-\langle w\rangle )\rangle$ is symmetric and has elements

(20) $$\begin{array}{l} \Sigma_{11}=\mu t+\frac{1}{8N_{e}\kappa }\left(1-e^{-8N_{e}\kappa \mu t}\right),\\ \Sigma_{12}=-\mu t+\frac{1}{8N_{e}\kappa }\left(1-e^{-8N_{e}\kappa \mu t}\right). \end{array}$$

The variance in the hybrid binding energies is due to a pure diffusive term μt, which represents how the two lineages diffuse apart by independent mutations, plus a term which represents the saturating growth of variance of each lineage after divergence due to the constraint provided by the free fitness landscape; at short times, $\Sigma_{11}\sim 2\mu t$, where an extra μt comes from the intra-lineage exploration of binding energies which is effectively diffusive at short times. For short times ($8N_{e}\kappa \mu t\ll 1$), the off-diagonal terms are zero, meaning that the binding energies of the two hybrids are uncorrelated. However, for long times ($8N_{e}\kappa \mu t\gg 1$) they become anti-correlated, again due to the constraints imposed by the fitness landscape.

The average hybrid fitness is given by ${\langle F_{h}(t)\rangle =-\frac{1}{2}\kappa_{F}\langle w^{2}\rangle =-\frac{1}{2}\kappa_{F}(\Sigma_{11}+\langle w\rangle }^{2})$:

(21) $$\langle F_{h}(t)\rangle =F_{h}(0)-\frac{1}{2}\mu \kappa_{F}t-\frac{\kappa_{F}}{16N_{e}\kappa }(1-e^{-8N_{e}\kappa \mu t}).$$

We see that on short times $\langle F_{h}(t)\rangle \approx F_{h}(0)-\mu \kappa_{F}t$, so hybrid fitness decreases linearly with time.

The dynamics of the probability of a DMI for each hybrid, irrespective of whether the other hybrid has a DMI or not, is simply given by the probability that the hybrid fitness *F*_*h*_ falls below the threshold fitness $F^{⁎}=-\frac{1}{2}\kappa_{F}{\left(\xi^{⁎}\right)}^{2}$, or $|w|>\xi^{⁎}$:

(22) $$P_{I}(t)=1-\int_{-\xi^{⁎}}^{\xi^{⁎}}p(w,t)\;dw,$$

The variable *w* is given by the sum of a number of Gaussian processes, so p(w,t) itself must be Gaussian which is completely specified by its mean Eq. (19) and variance $\Sigma_{11}={\langle w^{2}\rangle -\langle w\rangle }^{2}$ (20). From Eq. (22), the probability of a DMI is then simply an integral of a Gaussian, which can be expressed in terms of complementary error functions:

(23) $$P_{I}(t)=\frac{1}{2}\mathrm{erfc}(\frac{\xi^{⁎}-\langle w\rangle }{\sqrt{2\Sigma_{11}}})+\frac{1}{2}\mathrm{erfc}(\frac{\xi^{⁎}+\langle w\rangle }{\sqrt{2\Sigma_{11}}}).$$

Note that both the average hybrid fitness and the probability of incompatibilities (Eq. (23)) are functions of dimensionless quantities such as μt, $4\kappa_{F}N_{e}$, and $F^{⁎}/\kappa_{F}$.

**Fig. 2:**
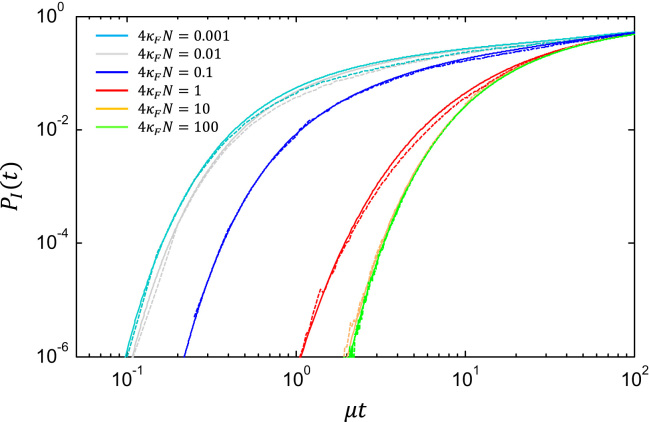
Log–log plot of the probability of a DMI *P*_*I*_(*t*) for a single hybrid as a function of time for fitness-scaled population sizes $4\kappa_{F}N_{e}=\{0.001,0.01,0.1,1,10,100\}$. Solid lines are the approximate analytical calculations using Eq. (23) and dotted lines are numerical integration of Eq. (15) using Eq. (22). The numerical simulations are split into two sets, one averaged over 10^4^ independent realisations extending to μt=1000 and one averaged over 10^6^ independent realisations extending to μt=1. The latter simulations are required to reach the smaller probabilities of an incompatibility. (For interpretation of the references to colour in this figure caption, the reader is referred to the web version of this paper.)

Eq. (23) represents a very different functional form for the growth of DMIs compared to that of Orr (Orr, 1995; Orr and Turelli, 2001), who predicted a power law form with divergence time. Here the growth of DMIs at short times has the form $P_{I}(t)\sim \mathrm{erfc}(1/\sqrt{t})\sim \sqrt{t}e^{-1/t}$ which has an essential singularity for *t*=0 and thus does not have a Taylor series expansion about *t*=0. The Laurent series in negative powers of *t* only becomes exact for small *t* with an infinite number of terms and when t≠0. As seen in Fig. 2, this means that on a log–log plot at small times *P*_*I*_(*t*) can never be approximated by a straight line and always has a negative curvature. This form arises since a finite time is required for hybrids to diffuse to the region of incompatibility ($|\xi |>\xi^{⁎}$).

## 5 Results

All results shown below assume an effective sequence length ℓ=10, $F^{⁎}/\kappa_{F}=-25$ ($\xi^{⁎}=\sqrt{50}\approx 7$) and $\xi (0)=\xi_{0}=1/2\kappa N_{e}$, which is the most probable value of *ξ* in equilibrium. Fig. 2 shows the probability of an incompatibility, for various values of $4\kappa_{F}N_{e}$, where solid lines are the analytical calculation and the dotted lines are from the numerical integration of Eq. (15), where no approximation is made regarding the values of *x*_1_ and *x*_2_. Firstly, we see that the analytical predictions compare well to integrating the full stochastic differential equations, validating our simplifying assumptions. Secondly, we see our coarse-grained continuous model predicts that there is a large population size effect for the probability of an incompatibility, where the characteristic time for incompatibilities to arise becomes much shorter as the population size decreases. In addition, it predicts that the dynamics of *P*_*I*_(*t*) become insensitive to differences in population size both for small population sizes ($\kappa_{F}N_{e}\ll 1/\ell$) and large population sizes ($\kappa_{F}N_{e}\gg 1/\ell$). Note that, as we have not bounded the Hamming distance to a maximum value of ℓ, *P*_*I*_ continues to increase at longer times (μt≪1); the limitations due to finite sequence length could be approximated by including additional sequence entropic potentials between hybrids in Eq. (11). However, it is the short time limit that is most relevant to speciation: if only a single DMI is needed for reproductive isolation, and there is a large number ($M\sim 10^{5}$) of pair-wise interacting loci in a genome, speciation would likely occur when $P_{I}\sim 1/M\sim 10^{-5}$.

**Fig. 3:**
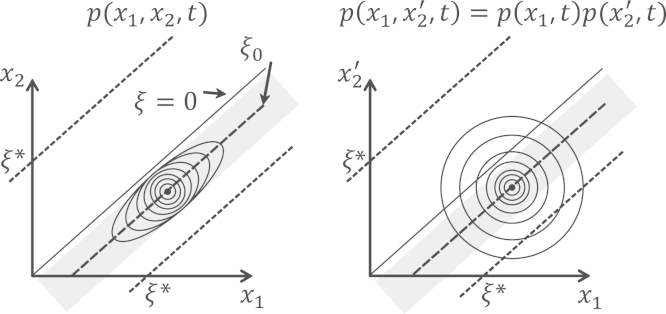
Evolution of $p(x_{1},x_{2},t)$ (left) and $p(x_{1},x_{2}^{\prime },t)$ (right) shown schematically, assuming the approximate single-peaked free fitness landscape $-\frac{1}{2}\kappa_{F}{\left(x_{1}-x_{2}-\xi_{0}\right)}^{2}$ (indicated by the dotted lines in Fig. 1B). Contours represent values of variables for some fixed arbitrary value of probability and how these contours move outwards with time. Variables *x*_1_ and *x*_2_ co-evolve and variables $x_{1}^{\prime }$ and $x_{2}^{\prime }$ co-evolve ($x_{1}^{\prime }$ and $x_{2}^{\prime }$ not shown) within the constraints set by the free fitness landscape on each lineage, while the hybrid density is a product of the marginal probability densities ($p(x_{1},x_{2}^{\prime },t)=p(x_{1},t)p(x_{2}^{\prime },t)$) and evolves in a spherically symmetric manner into the regions of incompatibility.

To understand this general behaviour, we can consider what happens to the time-dependent probability density of *x*_1_ and *x*_2_ for the first lineage, versus *x*_1_ and $x_{2}^{\prime }$ for the one of the hybrids, as shown in Fig. 3 (The probability density for $x_{1}^{\prime }$ and $x_{2}^{\prime }$ and $x_{1}^{\prime }$ and *x*_2_ are equivalent.). In the plots the lines represent some arbitrary contour of probability and how it changes with time. Initially, both lineage and hybrid populations diffuse neutrally and equally in all directions (variance ~2μt) up to the time $\sim {\left(2N_{e}\kappa \mu \right)}^{-1}$, when the change in free fitness is of order the mean fitness $\sim 1/4N_{e}$ and the accumulated variance of the parental lineages approaches the characteristic width of the potential Δξ (Eq. (14)). After this time the co-evolutionary constraint of the free fitness landscape is felt on each lineage and the probability density is then squeezed along a tube whose axis is defined by $x_{1}=x_{2}+\xi_{0}$ (assuming an initial condition $x_{1}(0)>x_{2}(0)$) and width Δξ. The tube of probability density is effectively squeezed from below by the requirement that populations on each lineage maintain good fitness, and from above by the constraint of sequence entropy, preventing populations from exploring unlikely phenotypes. As the marginal probability density for *x*_1_ and $x_{2}^{\prime }$ will be identical, in the hybrid phase-space, $p(x_{1},x_{2}^{\prime },t)$ are not affected by the inviability constraint and will continue to grow equally in all directions; incompatibilities arise when hybrid populations have diffused to one or the other critical binding energy at $x_{1}-x_{2}^{\prime }=\pm \xi^{⁎}$. From Eq. (23) and Fig. 3, we see that there will in general be two characteristic times for DMIs to arise, given by ${\left(\xi^{⁎}-1/2\kappa N_{e}\right)}^{2}\sim \Sigma_{11}(t)$ and ${\left(\xi^{⁎}+1/2\kappa N_{e}\right)}^{2}\sim \Sigma_{11}(t)$; however, the contribution of DMIs due to this second path will not be important at short times.

It is now simple to see where the populations size dependence of the growth of DMIs arises. As seen from Fig. 1A, at a given population size the balance between fitness and sequence entropy occurs for different values of binding energy; in other words at lower population sizes there is an increased probability for the acceptance of slightly deleterious mutations so that the balance between this tendency and selection leads to less fit equilibrium phenotypes. This means that the initial value of *ξ*_0_ is closer to the inviability boundary $\xi^{⁎}$ for smaller populations and so *ξ* and $\xi^{\prime }$ have to change less in order to produce Dobzhansky–Muller incompatibilities. For very small and very large populations, the value of *ξ*_0_ saturate at ℓ/2 and 0 respectively, representing the domination by sequence entropy and fitness, giving rise to an independence of speciation rate on the population size in these limits. At a sufficiently large population size, however, we might expect the continuous prediction to fail as populations probe the discrete changes in fitness due to mutations, causing a slow down of the substitution rate on each lineage and the rate of speciation.

## 6 Discussion and conclusion

Although the question of the population size dependence of speciation has received little empirical attention, there is indirect evidence from field data (Santos and Salzburger, 2012; Glor et al., 2004; Owen et al., 1990; Mayr, 1970, 1954; Rubinoff and Rubinoff, 1971; Fitzpatrick, 2004), as well as phylogenetic analyses (Coyne and Orr, 2004; Nee, 2001; Barraclough and Nee, 2001) and comparison of rates of developing reproductive isolation (Stelkens et al., 2010; Cooper and Penny, 1997) that smaller populations tend to speciate more quickly. Many of these traits are likely to involve only a few loci (Orr, 2001) and so evolve in the weak mutation, monomorphic regime; however, until recently, there have been no strong theoretical candidates to explain this speciation trend in the low mutation rate monomorphic regime, aside from founder event or peak-shift models that require a fine tuning of population reduction and growth on a fitness landscape with valleys.

In this paper, we address this question with a coarse-grained model of sequence evolution within a stochastic dynamics framework; this allows modelling at the continuous phenotypic level, where selection acts, while accounting for the fact that variation arises from mutations in sequences. The approach requires calculation of the sequence entropy of the genotype–phenotype map, which is the (log) number of sequences corresponding to a given phenotypic value. Although a master equation with this entropy function can be written down, in most cases it will be intractable, and require simulation; instead here we take the continuous limit of this master equation, which as shown rigorously in the Appendix, gives a Smoluchowski equation for the time-varying probability distribution of the phenotype, which is a diffusion equation where the mean change in phenotype per unit time is due the gradient of a potential function. The potential, or Lyapunov, function of evolution in the weak mutation regime is a sum of the fitness of phenotypes as well as the sequence entropy weighted by the inverse of the population size (Eq. (2)) and termed the free fitness (Iwasa, 1988). Our results predict that smaller populations develop hybrid incompatibilities more quickly. This can be understood with a simple picture of how incompatibilities arise; on each lineage protein and regulatory DNA sequences co-evolve within a free fitness landscape with a balance between good binding affinity and high sequence entropy, yet the hybrid binding affinities diffuse neutrally in a manner independent of population size. As the effect of sequence entropy is stronger at smaller population sizes, the common ancestor populations are poised closer to incompatible regions and so hybrids have a smaller phenotypic distance to diffuse, giving rise to a higher rate of speciation.

The coarse-grained model predicts that this population-size effect diminishes for large population sizes $4\kappa_{F}N_{e}\gg 1$, as fitness dominates and the phenotypic distance needed to diffuse does not change; however, in reality, since *κ*_*F*_ is roughly the scale of fitness differences for discrete sequence evolution, this is also the regime where evolution on each lineage can feel the discreteness of the changes in fitness and the substitution rate diminishes. This may lead to an additional mechanism that slows down the rate of speciation as the population size increases; this is not seen by the low population size effective coarse-grained theory presented in this paper. In this respect, real TF–TFBS pairs at large population sizes ($4\kappa_{F}N_{e}\gg 1$) likely share a similarity to incompatibilities in polygenic traits (Barton, 1989), since in both cases optima are separated by deleterious intermediates, causing a slow down of divergence with increasing population size. A simple way to account for this discreteness in the coarse-grained theory is to replace the mutation rate with the average substitution rate expected from discrete sequence evolution. Another way to account for this heuristically in a coarse-grained theory would be to introduce a characteristic phenotypic scale of mutations, below which the dynamics becomes frozen, for example, by introducing terms proportional to the time-derivative of the curvature of the probability distribution in the Smoluchowski equation (Khatri and McLeish, 2007). Although this theory strictly applies to the monomorphic regime, we would also expect the effect of sequence entropy to lead to a similar trend of an increasing rate of speciation for decreasing population size for polymorphic loci; we expect the effects due to sequence entropy and discreteness of fitness differences would be reinforced by the slowed divergence of allopatric lineages due to the mechanism of Gavrilets (1999) and Nei et al. (1983), where highly diverged members tend to produce a higher fraction of inviable offspring. In particular, recent sequence-level simulations have shown indications that such a population size effect also exists in the intermediate strength regime of mutations $4N_{e}\mu \sim 1$, although the growth of DMIs with divergence time was not investigated in detail (Tulchinsky et al., 2014).

Finally, the biophysical model of speciation we present provides a very different picture on how incompatibilities develop compared to the Orr model (Orr, 1995; Orr and Turelli, 2001). Hybrid populations have to diffuse a finite distance and so there is a latency in the development of incompatibilities; mathematically, this gives rise to a non-polynomial functional form for the growth of DMIs, in contrast to the polynomial increase suggested by Orr. In particular, this manifests itself as a negative curvature for small times in a log–log plot of the number or probability of DMIs versus time. However, we note that this result assumes diffusion from a fixed common ancestral phenotypic state (assumed here for simplicity to be the most probable), whereas in reality, there will be a range of phenotypic values drawn from the equilibrium distribution, potentially changing this law of growth of DMIs, particularly at small population sizes, where the common ancestor distribution is broad. The alternative hypothesis for the growth of DMIs we present may be tested with more detailed studies of species divergence at different population sizes, similar to current works (Matute et al., 2010; Moyle and Nakazato, 2010) which show a rapid increase of hybrid incompatibilities with divergence, which given the paucity of data points is consistent with both the Orr model and the one presented here. In particular, recent cross-species ChiP-seq analysis of transcription factor binding (Schmidt et al., 2010) suggests a way to explicitly test our predictions at the level of actual binding affinities of hybrid TF–TFBS combinations for recently diverged species.

Gene expression divergence is thought to underly many differences between species (King and Wilson, 1975; Wolf et al., 2010; Wray, 2007), for example, in the Galapagos finches (Abzhanov et al., 2006), the various species of *Drosophila* (Wittkopp et al., 2008) and with more direct evidence of a role in speciation through the evolution of genes related to transcription factors (Ting et al., 1998; Brideau et al., 2006). Protein binding DNA to control gene expression is a prototypical co-evolving system and critical for the proper development of organisms; here we have explored a realistic coarse-grained stochastic dynamics approach to modelling phenotypic change that incorporates, through an appropriate sequence entropy function, the effects of mutations on protein and DNA sequences. We suggest that such a coarse-grained approach will allow tractable modelling of more complicated gene regulatory systems and thus provide insight on their evolution and their role in speciation. Finally, although we have studied TF–TFBS binding, molecular recognition between two sequences arises in many different biological contexts, such as antibody–antigen binding, protein–protein interactions and the interaction between genes expressed in the nucleus and mitochondria and we expect our results to have relevance to these systems.

## Appendix A Kramers–Moyal expansion of a generalised master equation for sequence driven phenotypic evolution

In this appendix, we derive the one-dimensional Smoluchowski equation for phenotypic evolution with sequence entropic effects, starting from a master equation for the probability distribution of fixed phenotypes. The general master equation for a phenotypic variable *ξ* is

(A.1) $$\frac{\partial p(\xi ,t)}{\partial t}=\int d\xi^{\prime }(W(\xi |\xi^{\prime })p(\xi^{\prime },t)-W(\xi^{\prime }|\xi )p(\xi ,t)).$$

We make the assumption that a generalised genotype–phenotype map has the property that mutations give rise to transitions in phenotype that are local, such that the rate *W* is of the form

(A.2) $$W(\xi^{\prime }|\xi )=W_{+}(\xi )\delta_{\xi^{\prime },\xi +\delta \xi }+W_{-}(\xi )\delta_{\xi^{\prime },\xi -\delta \xi }.$$

where δξ is the size of discrete jumps and δξ=1 would represent, for example, the Hamming distance between two sequences; we are interested in taking the continuous limit of Eq. (A.1), as δξ→0. The Kramers–Moyal expansion is simply a Taylor expansion about the state *ξ* in the jump size δξ and retains the first and second order terms, assuming that these will be most important as δξ→0. This in general gives rise to a non-linear Fokker–Planck equation. With the rates *W* in this form, the resulting Fokker–Planck equation is (Gardiner, 2009; van Kampen, 1981)

(A.3) $$\frac{\partial p(\xi ,t)}{\partial t}=-\frac{\partial }{\partial \xi }(\alpha_{1}(\xi )p(\xi ,t))+\frac{1}{2}\frac{\partial^{2}}{\partial \xi^{2}}(\alpha_{2}(\xi )p(\xi ,t))$$

where the functions *α*_1_ and *α*_2_ are the 1st and 2nd order moments of the transition rates, respectively:

(A.4) $$\alpha_{n}(\xi )=\int d\xi^{\prime }{\left(\xi^{\prime }-\xi \right)}^{n}W(\xi^{\prime }|\xi )=\delta \xi^{n}(W_{+}(\xi )+{\left(-1\right)}^{n}W_{-}(\xi )).$$

We now make the assumption that the transition rates are of the form

(A.5) $$W_{\pm }\left(\xi \right)=2\mu_{0}N_{e}n_{\pm }\left(\xi \right)\pi \left(F\left(\xi \right),F\left(\xi \pm \delta \xi \right)\right),$$

where *μ*_0_ is the mutation rate per site, *N*_*e*_ is the effective population size, $n_{+}$ is the number of mutational paths that increase the phenotype from ξ→ξ+δξ and $n_{-}$ is the mutational paths that decrease the phenotype from ξ→ξ−δξ, with the constraint that $n_{+}+n_{-}=n$, i.e. the total number of mutational paths is fixed to the total number of sites *n*.

We assume the fixation probability *π* is of the Kimura (1962) form:

(A.6) $$\pi (F(\xi ),F(\xi \pm \delta \xi ))=\frac{1-e^{-2(F(\xi \pm \delta \xi )-F(\xi ))}}{1-e^{-4N_{e}(F(\xi \pm \delta \xi )-F(\xi ))}}.$$

We are interested in the form of the rate, and hence the fixation probability, in the limit that δξ→0, for which we expect that to first order $F(\xi \pm \delta \xi )\approx F(\xi )\pm \delta \xi \partial_{\xi }F(\xi )=F(\xi )\pm \delta F(\xi )$. In this limit π(F(ξ),F(ξ±δξ))=π(±δF), which can be expanded to first order in δF as

(A.7) $$\pi (\pm \delta F)\approx \frac{1}{2N_{e}}(1\pm 2N_{e}\delta F).$$

To calculate the dependence of the moments on the change in entropy, we note that the ratio of the forward and backward mutational paths will be equal to the ratio of the number of states for ξ±δξ, assuming that in the limit that δξ→0, the number of states varies slowly:

(A.8) $$\frac{n_{+}}{n_{-}}=\frac{\Omega (\xi +\delta \xi )}{\Omega (\xi )}=\frac{\Omega (\xi )}{\Omega (\xi -\delta \xi )}=e^{\delta S}.$$

Given $n_{+}+n_{-}=n$, it is then simple to show that

(A.9) $$\delta S\approx 2(n_{+}-n_{-})/n.$$

Plugging these results into Eq. (A.4) for the 1st and 2nd moments, we find

(A.10) $$\alpha_{1}(\xi )=2\mu_{0}{\mathrm{nN}}_{e}\delta \xi (\delta F+\frac{\delta S}{4N_{e}})=2\mu_{0}{\mathrm{nN}}_{e}\delta \xi \delta \Phi .$$

and

(A.11) $$\alpha_{2}(\xi )=n\mu_{0}{\left(\delta \xi \right)}^{2}(1+N_{e}\delta F\delta S)\approx n\mu_{0}{\left(\delta \xi \right)}^{2},$$

where in *α*_*2*_ we ignore the 2nd term as it will be of order ${\left(\delta \xi \right)}^{2}$. Then taking the limit that δξ→0, such that the combination $n\mu_{0}{\left(\delta \xi \right)}^{2}\to \mu$ remains finite, and $\lim_{\delta \xi \to 0}\delta \Phi /\delta \xi \to \partial_{\xi }\Phi$ we arrive at the Smoluchowski equation:

(A.12) $$\frac{\partial p}{\partial t}=\frac{1}{2}\mu \frac{\partial }{\partial \xi }(\frac{\partial p}{\partial \xi }-4N_{e}p\frac{\partial \Phi }{\partial \xi }).$$

Our particular choice of the effective diffusion constant of the dynamics as μ/2 means that *μ* represents the overall mutation rate for all base-pairs, *n*, in the loci involved; in particular, this means that the overall dynamics of results from discrete simulations will agree with solutions of the continuous diffusion theory, for example, represented by Eqs. (A.12) and (6).

## Footnotes

1 This latter term would be called the *drift* in the physics literature, which we avoid here and reserve this name in association with *genetic drift*.

2 As discussed above, we would expect that in the long time limit, $\langle w\rangle =\langle w^{\prime }\rangle =0$, as $x_{1}-x_{2}^{\prime }$ and $x_{1}^{\prime }-x_{2}$ will be equally likely to positive as negative given the co-evolution of $x_{1}-x_{2}$ and of $x_{1}^{\prime }-x_{2}^{\prime }$ on the bistable free fitness landscape shown in Fig. 1B; however, as the analytical approximation only considers evolution of $x_{1}-x_{2}$ and $x_{1}^{\prime }-x_{2}^{\prime }$ on the single-peaked landscape shown by the dotted line of Fig. 1B, the averages of *w* and $w^{\prime }$ will be *ξ*_0_, since this is the value of the binding energy for the common ancestor. The accuracy of the analytical results for short times (μt⪯¡1) is demonstrated below.
